# The Upscale Manufacture of Chondrocytes for Allogeneic Cartilage Therapies

**DOI:** 10.1089/ten.tec.2023.0037

**Published:** 2023-09-14

**Authors:** Charlotte H. Hulme, John K. Garcia, Claire Mennan, Jade Perry, Sally Roberts, Kevin Norris, Duncan Baird, Larissa Rix, Robin Banerjee, Carl Meyer, Karina T. Wright

**Affiliations:** ^1^ Centre for Regenerative Medicine Research, School of Pharmacy and Bioengineering, Keele University, Keele, Staffordshire, United Kingdom.; ^2^ Robert Jones and Agnes Hunt Orthopaedic Hospital, Oswestry, Shropshire, United Kingdom.; ^3^ TeloNostiX Ltd, Central Biotechnology Services, Cardiff, United Kingdom.; ^4^ School of Medicine, Cardiff University, Cardiff, Wales, United Kingdom.

**Keywords:** chondrocytes, hollow-fiber bioreactor, large-scale expansion, allogeneic cell therapy, cartilage cell repair, human platelet lysate, fetal bovine serum

## Abstract

Allogeneic chondrocyte therapies need to be developed to allow more individuals to be treated with a cell therapy for cartilage repair and to reduce the burden and cost of the current two-stage autologous procedures. Upscale manufacture of chondrocytes using a bioreactor could help provide an off-the-shelf allogeneic chondrocyte therapy with many doses being produced in a single manufacturing run. In this study, we assess a good manufacturing practice-compliant hollow-fiber bioreactor (Quantum^®^) for adult chondrocyte manufacture. Chondrocytes were isolated from knee arthroplasty-derived cartilage (*n* = 5) and expanded in media supplemented with 10% fetal bovine serum (FBS) or 5% human platelet lysate (hPL) on tissue culture plastic (TCP) for a single passage. hPL-supplemented cultures were then expanded in the Quantum bioreactor for a further passage. Matched, parallel cultures in hPL or FBS were maintained on TCP. Chondrocytes from all culture conditions were characterized in terms of growth kinetics, morphology, immunoprofile, chondrogenic potential (chondrocyte pellet assays), and single telomere length analysis. Quantum expansion of chondrocytes resulted in 86.4 ± 38.5 × 10^6^ cells in 8.4 ± 1.5 days, following seeding of 10.2 ± 3.6 × 10^6^ cells. This related to 3.0 ± 1.0 population doublings in the Quantum bioreactor, compared with 2.1 ± 0.6 and 1.3 ± 1.0 on TCP in hPL- and FBS-supplemented media, respectively. Quantum- and TCP-expanded cultures retained equivalent chondropotency and mesenchymal stromal cell marker immunoprofiles, with only the integrin marker, CD49a, decreasing following Quantum expansion. Quantum-expanded chondrocytes demonstrated equivalent chondrogenic potential (as assessed by ability to form and maintain chondrogenic pellets) with matched hPL TCP populations. hPL manufacture, however, led to reduced chondrogenic potential and increased cell surface positivity of integrins CD49b, CD49c, and CD51/61 compared with FBS cultures. Quantum expansion of chondrocytes did not result in shortened 17p telomere length when compared with matched TCP cultures. This study demonstrates that large numbers of adult chondrocytes can be manufactured in the Quantum hollow-fiber bioreactor. This rapid, upscale expansion does not alter chondrocyte phenotype when compared with matched TCP expansion. Therefore, the Quantum provides an attractive method of manufacturing chondrocytes for clinical use. Media supplementation with hPL for chondrocyte expansion may, however, be unfavorable in terms of retaining chondrogenic capacity.

**Impact statement:**

This is the first study, to our knowledge, to manufacture adult chondrocytes in a good manufacturing practice-compliant hollow-fiber bioreactor (Quantum^®^). We provide evidence that chondrocytes can be manufactured using this methodology while retaining comparable properties to chondrocytes expanded in matched tissue culture plastic conditions. Upscale Quantum^®^ expansion may provide an appropriate method for developing allogeneic chondrocyte therapies.

## Introduction

The use of autologous chondrocyte implantation (ACI) to treat chondral/osteochondral defects has been applied clinically for nearly 30 years.^1^ Despite this, advancement of such cell therapies is needed to reduce their cost and to improve their availability to a wider patient population. Currently, the production of autologous chondrocytes as advanced therapy medicinal products (ATMPs) is laborious and costly due to the need to produce a cell product for each patient. Furthermore, chondrocyte therapies are limited by the number of cells that can be expanded within a limited time frame using traditional tissue culture plastic (TCP) methods. Currently, multiple (2–3) passages are required to achieve the numbers of cells used to treat small defects,^2^ and thus, treatment of multiple or large chondral/osteochondral lesions is often not possible, restricting the option of this therapy for some patients.

The move from autologous to allogeneic chondrocyte therapy has the potential to produce large quantities of homogenous cells that can be cultured from “optimal” donors, selected for their therapeutic potential, to produce an “off-the-shelf” bank of multiple doses. Along with the beneficial cost implications, this would allow for a “single-stage” surgical procedure as opposed to the current ACI treatment in which the patient undergoes two surgeries (the first to harvest a cartilage biopsy and the second to implant the patients' culture-expanded chondrocytes). In order for allogeneic chondrocyte therapy to become a reality, a good manufacturing practice (GMP)-compliant process in which large numbers of high-quality chondrocytes can be cultured is needed.

We have previously demonstrated that use of the Quantum^®^ bioreactor (manufactured by Terumo BCT, Lakewood, USA) can generate large numbers of bone marrow- and umbilical cord-derived mesenchymal stromal cells (MSCs), which maintain their phenotypic properties following rapid expansion.^3^ This bioreactor system has been widely used to expand human MSCs from various tissue sources, including production of cells used clinically, as we have reviewed and illustrated in Hulme et al.^4^ We are, however, unaware of any studies that have culture-expanded chondrocytes using this platform. The Quantum^®^ cell expansion system comprised hollow fibers that provide a surface area of 2.1 m^2^ for cell adherence, equivalent to 120 T175 flasks used in standard tissue culture practice. The Quantum^®^ is GMP-compliant, having been used in multiple clinical trials.^4^

All the constituent parts of the bioreactor are produced to a safety and quality standard, with relevant documentation and traceability such that the European Medicine Agency approve the use for GMP.^5^ For expansion of adherent cells, the polysulfone fibers are lined with a thin surface coating of a substrate to allow for the attachment of a monolayer of cells. Once cells are adhered to the fibers, culture media are continuously perfused through the fibers and the cell growth is assessed through monitoring of cellular metabolism by measuring the secretion of lactate into and also the consumption of glucose from the conditioned media. The flow rate at which media are perfused over the cells is altered as the cell numbers increase, to maximize cell expansion.

In the ACI procedure, chondrocytes are expanded in media supplemented with autologous serum collected from patients by venipuncture at the time of initial surgery for cartilage harvest.^2,6^ For large-scale allogeneic cultures, this would not represent a viable serum source. Despite clinical-grade fetal bovine serum (FBS) currently being used in the culture of GMP-compliant ATMPs, identification of xeno-free alternatives to FBS is needed to improve the safety profile of these products while adhering to regulatory policies.^7^ The use of pooled human platelet lysate (hPL) provides an attractive alternative, which is manufactured to GMP standards and without the risk of xenogeneic reaction or transmission of bovine pathogens.^8^ Akin to FBS, hPL is rich in vitamins, minerals, cytokines, and growth factors and has been demonstrated to support the culture of numerous cell types, particularly MSCs.^9^ However, the culture of chondrocytes in FBS remains the “gold standard” for research purposes against which alternative serum sources should be compared.^9^

In this study, we have assessed the potential of the Quantum^®^ bioreactor as a platform for the large-scale culture expansion of chondrocytes. This has been undertaken using a two-step “hybrid” process in which chondrocytes were isolated and expanded via standard protocols using TCP^2,10^ in culture media supplemented with either 10% FBS or 5% hPL (Stemulate™; Cook Regentec, Indianapolis, USA). Those chondrocytes culture expanded in 5% hPL were either seeded in the Quantum^®^ or onto TCP (again in 5% hPL), and 10% FBS-cultured cells were seeded onto TCP (in 10% FBS) for the second expansion phase. The need for a hybrid process allows for the limited number of chondrocytes that can be isolated to be culture expanded such that a sufficient number can be seeded into the large surface area of the Quantum^®^ bioreactor.

We have carried out a comprehensive characterization of the Quantum^®^-expanded chondrocytes and compared them with parallel cultures on TCP both using hPL and FBS, determining whether these cells maintain their chondrogenic phenotype via assessment of a panel of chondropotency markers and chondrogenesis assays, as well as evaluating the effect of rapid expansion in relation to telomere length distributions.

## Methods

### Patients

Cartilage samples were collected with informed consent from five patients undergoing total knee replacement (TKR) surgery for the treatment of end-stage osteoarthritis (OA). Ethical approvals from the National Research Ethics Service- North West Committee (11/NW/0875) were in place. The individual donors were aged 56–71 and there were four females and one male (Table 1).

**Table 1.** Demographics of Patients Undergoing Total Knee Replacement Surgery from Which Cartilage Was Harvested

Donor number	Gender	Age
1	Male	56
2	Female	71
3	Female	61
4	Female	65
5	Female	70

### Chondrocyte isolation and expansion

Femoral condyles from patients undergoing TKR surgery were assessed for areas of macroscopically normal articular cartilage. Maintaining sterile conditions, this full-depth cartilage was excised, weighed, and minced into small pieces, which were then digested for 16 h at 37°C using collagenase (250 IU/mg dry weight; Worthington, New Jersey, USA) in serum-free Dulbecco's modified Eagle's medium/F-12 (DMEM/F-12; Life Technologies, Paisley, UK). Following digestion, the media/cell suspension was strained using a 40 μm cell strainer and then centrifuged at 350 *g* for 10 min to produce a cell pellet that was reconstituted in 1 mL media. Following a cell count, ∼75% of the cells were seeded on TCP (Sarstedt, Leicester, UK) in DMEM/F-12 with 1% (v/v) penicillin/streptomycin (P/S; Life Technologies) and 5% hPL (Stemulate^®^; Cook Regentec), hereafter referred to as hPL-medium. hPL was used from a commercial source, Cook Regentec, which produced hPL to a GMP-compliant standard.

In all manufacturing runs, the same batch of hPL was used to minimize the influence between donors. The proportion of hPL in the media (5%) was used based on recommendations from the supplier and as this is the most widely published concentration in studies looking to move away from FBS.^11^ Seeding of cells into the hPL was prioritized to ensure there were sufficient cells at P0–1 to seed into the Quantum^®^ bioreactor. The remaining cells were seeded in DMEM/F-12 with 1% P/S and 10% FBS (Life Technologies), hereafter referred to as FBS-medium. The FBS-medium represents the typical chondrocyte culture medium, where 10% FBS is typically used as a “gold standard” comparator.^6^

All donor samples were maintained separately (nonpooled) and expanded in hPL or FBS (i.e., to compare matched donor expansion in the different supplements on TCP or in the Quantum^®^). Chondrocytes were seeded at 5 × 10^3^ cells/cm^2^ and were maintained in a humidified atmosphere at 37°C with 21% O_2_ and 5% CO_2_, changing the media every 2–3 days. This culture expansion phase is deemed passage 0 (P0).

Once the chondrocytes cultured in hPL-medium reached 70–80% confluence, they were trypsinized and seeded into the Quantum^®^ or reseeded onto TCP at 5 × 10^3^ cells/cm^2^ in hPL-medium (deemed passage 1 [P1]). The numbers of chondrocytes seeded into the Quantum^®^ or onto TCP are detailed in Table 2. Chondrocytes initially seeded onto TCP in FBS-medium were trypsinized at 70–80% confluence and then reseeded onto TCP at 5 × 10^3^ cells/cm^2^ in FBS-medium (P1).

**Table 2.** Passage 0 and Passage 1 Cell Growth Characteristics of Chondrocytes in the Quantum and on Tissue Culture Plastic in Human Platelet Lysate and Fetal Bovine Serum

		Cells seeded ( × 10^6^)	Cells harvested ( × 10^6^)	Increased cell yield ( × 10^6^)	Days in culture	Population doublings
P0–1	TCP-human platelet lysate	4.1 ± 0.4 (3.5–4.4)	13.0 ± 4.9 (5.4–18.7)	8.9 ± 4.5 (1.9–14.3)	13.2 ± 1.3 (11–14)	
	TCP-fetal bovine serum	2.0 ± 1.3 (0.4–3.5)	6.3 ± 5.4 (0.4–13.6)	4.4 ± 4.1 (0.03–10.1)	16.0 ± 4.5 (11–23)	
P1–2	TCP-human platelet lysate	1.4 ± 0.7 (0.4–2.1)	5.9 ± 4.4 (2.2–13.2)	4.5 ± 3.7 (1.8–11.4)	7 ± 2.7 (3–10)	2.1 ± 0.6 (1.5–2.9)
	TCP-fetal bovine serum	0.13 ± 0.93 (0.4–2.6)	3.8 ± 3.0 (1.5–8.2)	2.6 ± 3.2 (0.3–7.3)	7.7 ± 4.2	1.8 ± 1.3 (0.1–3.2)
	Quantum^®^-human platelet lysate	10.2 ± 3.6 (5.0–15.0)	86.4 ± 38.5 (24.0–120.0)	76.2 ± 34.9 (14.0–109.2)	8.4 ± 1.5 (7–10)	3.0 ± 1.0 (1.3–3.9)

Data are mean ± SD, with the range displayed in brackets (*n* = 5).

P, passage; TCP, tissue culture plastic.

### The Quantum cell expansion system

The Quantum cell expansion system was prepared as described previously.^3^ Briefly, the system was precoated overnight with 100 mL of pooled human cryoprecipitate from five donors (NHS Blood and Transplant, Birmingham, UK) diluted 1:1 (v/v) with PBS. This coating allows for the adherence of cells to the polysulfone hollow fibers. The system was conditioned with hPL-media, after which 5–10 × 10^6^ chondrocytes were seeded into the Quantum cell expansion system and were left to adhere with uniform suspension for 24 h. The Quantum cell expansion system maintained perfusion of the hPL-medium over the cells while removing an equal volume of conditioned medium. The concentration of lactate and glucose within the conditioned medium was assessed daily using a Lactate Plus meter (Nova Biomedical, Runcorn, UK) and a clinical blood glucose meter (Kinetik Wellbeing, Redhill, UK), respectively.

The lactate and glucose concentrations served as indicators of cellular metabolism and consequently as a proxy of cell number. As the number of cells within the system increased, the perfusion rate of fresh medium was increased from a baseline rate of 0.1 to 1.6 mL/min. Once a flow rate of 1.6 mL/min was achieved, chondrocytes were cultured for a further 12–24 h before being harvested. Alternatively, chondrocytes were harvested if their growth rate was deemed to plateau, as assessed via conditioned medium lactate and/or glucose concentration. Chondrocytes were harvested using TrypLE™ (Gibco, New York, US), as this product can easily be switched to a GMP-compliant alternative should the process be adopted for ATMP manufacture.

Briefly, the preprogrammed harvest protocol from TerumoBCT was used in which the hollow fibers containing the cells were fully washed with PBS; TrypLE loaded into the fibers and held at 37°C for 8 min and then a matched volume of culture medium added and flushed out of the system into a cell harvest bag. This process was repeated twice to ensure that all cells were harvested. Harvested cells, in TrypLE and culture medium, were aliquoted into tubes before centrifugation at 400 *g* for 10 min to produce a cell pellet, which was reconstituted in 1 mL media.

### Calculation of growth kinetics

Doubling time (DT) was calculated using the following formula:

DT = (*t*_2_ − *t*_1_) × ln (2)/ln (*n*_2_/*n*_1_)

where *t*_1_ is the time at seeding, *t*_2_ is the time at harvesting, *n*_1_ is the cell number at seeding, and *n*_2_ is the cell number at harvest. To calculate the number of population doublings of chondrocytes, the following formula was used: DT = 3.32 × (log *N*_2_ − log *N*_1_), where *N*_1_ is the cell number at seeding and *N*_2_ is the cell number at harvest.

### Flow cytometry immunoprofiling of chondrocytes

Chondrocytes were harvested by trypsinization at P0 and P1 from TCP and/or the Quantum^®^. Cells were centrifuged, counted, and prepared at 20,000 cells per tube. The cells were blocked using 10% human IgG in 2% bovine serum albumin (BSA) and then resuspended in 2% BSA for flow cytometry. The following fluorochrome-conjugated antibodies were used to assess chondrogenic potency markers^10,12–16^: CD166-Brilliant Violet 421 (BV421) (clone 3A6), CD39-Allophycocyanin (APC) (clone TU66), CD44-Peridinin-chlorophyll proteins-Cyanine 5.5 (PerCP-Cy5.5) (clone G44-26) (all from Becton Dickinson and Company, Oxford, UK), and CD151-PE (clone14A2.H1) (R&D Systems, Abingdon, UK). Markers to indicate MSC profiles^17^ were assessed using the following antibodies: CD105-APC (clone 266), CD73-BV421 (clone AD2), CD90-Phycoerythrin (PE) (clone 5E10), CD19-BV421 (clone HIB19), CD45-PE (clone HI30), CD34-APC (clone 581), and CD14-PerCP-Cy5.5 (clone M*φ*P9) (all antibodies from Becton Dickinson and Company).

Antibodies used to assess integrin immunoprofiles were CD29-APC (MAR4), CD49a-PE (clone SR84), CD49b-BV421 (clone 12F1), CD49c-PE (clone C3 II.1), and CD151/61-PE (clone 23C6) (also all from Becton Dickinson and Company).

Chondrogenic potency was also assessed using an antibody to intracellular SOX-9 (clone 3C 10; Abcam, Cambridge, UK). Briefly, cells were fixed in 80% (v/v) methanol, permeabilized in 0.1% (v/v) Tween-20 in PBS, blocked in 0.1% (v/v) Tween-20 in PBS with 10% human IgG, and then resuspended in 0.1% (v/v) Tween-20 in PBS for flow cytometry.

Isotype-matched IgG controls were used in the gating strategy for all antibodies. Flow cytometry analysis was performed using an FACSCanto II flow cytometer using Diva 7 software (Becton Dickinson and Company).

### Chondrogenic differentiation assays

Chondrogenic pellet cultures were established following expansion in the Quantum^®^ or on matched TCP conditions. In brief, 2 × 10^5^ chondrocytes per pellet were centrifuged at 500 *g* for 8 min in chondrogenic differentiation media. This comprised 1% insulin–transferrin–selenium (Gibco™, Fisher Scientific, UK), 10 ng/mL transforming growth factor-β (PeproTech, USA), 1 mM ascorbic acid-2-phosphate, 10 μM dexamethasone, 20 μM linoleic acid, 1 mM sodium pyruvate (all Sigma Aldrich, UK), and 1% P/S (Life Technologies) made up in DMEM/F-12 (Life Technologies). Following 3 days in static culture, the chondrogenic pellets were dislodged from the Eppendorf tube. Media were changed on the pellets every 2–3 days and the pellets were maintained in culture for 28 days, then washed with PBS (Life Technologies), and snap-frozen in liquid nitrogen. Frozen chondrogenic pellets were stored at −80°C until subsequent analysis.

### Histological analysis of chondrogenic pellets

Chondrogenic pellets were cryosectioned (7 μm) using a cryostat (Bright Instrument Co Ltd, Huntingdon, UK) onto poly-L-lysine-coated slides. Slides were stained for glycosaminoglycans (GAGs) using the metachromatic stain, 1% aqueous toluidine blue (BDH) covering the slides for 30 s, and then washing in tap water. Following air-drying, slides were mounted in Pertex (Cell Path Ltd, Newtown, UK).

### GAG/DNA analysis of chondrogenic pellets

Chondrogenic pellets were digested in 125 μg/mL papain, made up in a buffer of 5 mM ethylenediaminetetraacetic acid, 5 mM cysteine hydrochloride, and 0.1 M sodium phosphate (all Sigma Aldrich) and adjusted to pH 6.5, for 3 h at 60°C. The pellets in buffered papain were vortexed every 30 min throughout the 3-h digest, to release GAGs and DNA. These samples were then centrifuged at 1000 *g* for 5 min and stored at −20°C for subsequent analysis.

Quantitative assessment of GAG concentration was performed to indicate the capacity of the chondrocytes to form extracellular matrix when driven toward chondrogenesis, in pellet assays. This method is widely used to provide an indication of the chondrocyte's cartilage forming potential and hence their likely capacity to repair damaged cartilage.^10,18–20^ GAGs were quantitated using the dimethyl blue (DMMB) assay.^21,22^ Bovine trachea-derived chondroitin sulfate (Sigma Aldrich) was used to prepare standards in PBS, with serial dilutions from 0 to 20 μg/mL. Fifty microliters of sample or standard and 200 μL of 4 × DMMB staining solution was combined per well of a 96-well plate. The assay absorbance was immediately read at 530 nm. The total GAG content for each sample was calculated using the equation of the linear portion of the standard curve.

A PicoGreen assay (Invitrogen, Massachusetts, USA) was used to quantitate the amount of double-stranded DNA in the papain-digested pellet solution. The assay was performed according to the manufacturer's instructions. Assay fluorescence was measured on a plate reader (Omega FLUROStar; BMG Labtech, Ortenberg, Germany) with excitation at 480 nm and emission at 520 nm.

The GAG content of each chondrogenic pellet was normalized to its DNA content, calculated by dividing the total GAG content by the DNA content of the same pellet.

### DNA extraction and single telomere length analysis

DNA was isolated from 3 × 10^5^ chondrocytes immediately after harvest from culture on TCP or in the Quantum in hPL-medium (P1). DNA was extracted using the High Pure PCR Template Preparation Kit (Roche, Sussex, UK). Extracted DNA was then stored at −80°C until the time of analysis. The DNA from matched donor chondrocytes expanded in the Quantum or on TCP was subjected to single telomere length analysis (STELA) at the 17p telomeres, as described previously.^23,24^

### Statistical analysis

Statistical analysis was performed using Prism software version 9.0 (GraphPad Software, CA, USA). The normality of the data was assessed using a Shapiro–Wilk test, which was used to inform whether parametric or nonparametric statistical tests were appropriate. Unpaired data were analyzed using an unpaired Student *t* test or Mann–Whitney U test, where appropriate, based on the normality of the data set. Paired data were analyzed using a paired *t*-test or a Wilcoxon-matched pairs signed rank test, where appropriate. For multiple comparisons, analysis of variance or Kruskal–Wallis was used, with either a Holm–Sidak or Dunn's multiple comparisons *post hoc* test, respectively, where appropriate. *p*-Values ≤0.05 were considered significant.

## Declarations

### Ethics approval and consent to participate

Patient samples were collected under the ethics approvals: “Investigating the potential for cells and molecules isolated from orthopaedic patients for modelling and understanding pathogenic conditions and developing diagnostic markers and therapies for musculoskeletal disorders and spinal cord injury” (11/NW/0875), which was approved by the NRES committee North West-Liverpool East. All patients provided valid informed consent before samples were collected.

## Results

### Chondrocyte growth and cell morphology

**FIG. 1.**
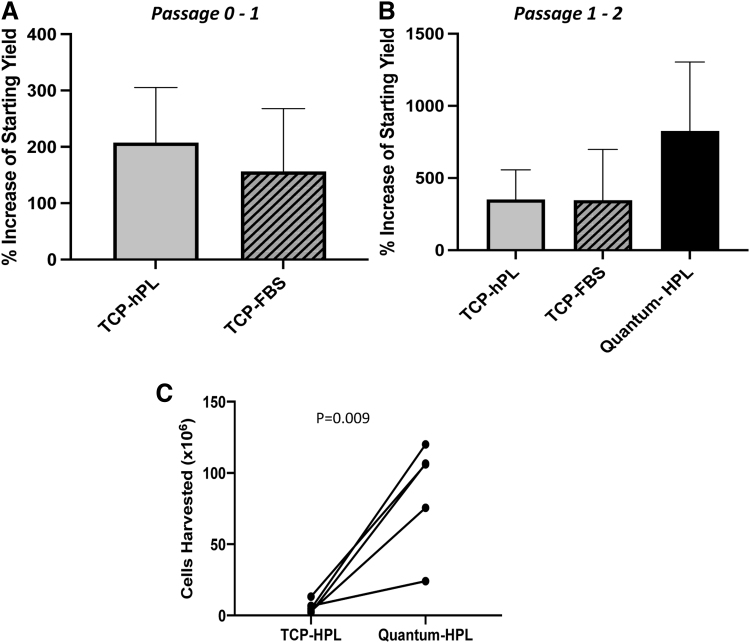
**(A)** The percentage increase in the number of chondrocytes harvested compared with the number seeded following initial passage (P0–P1) on TCP was not different between cells cultured in hPL cf. FBS (*p* < 0.05; paired *t*-test). **(B)** The percentage increase in chondrocytes following culture in the Quantum^®^ bioreactor was not significantly increased compared with on TCP in hPL or on TCP in FBS (*p* > 0.05; paired *t*-test). Data are mean ± standard deviation. **(C)** The number of chondrocytes harvested from matched donor populations was significantly higher following Quantum^®^ expansion compared with matched hPL expansion on TCP (*p* = 0.009; paired *t*-test). FBS, fetal bovine serum; hPL, human platelet lysate; TCP, tissue culture plastic.

Chondrocytes could be maintained and expanded in the Quantum^®^ bioreactor. At the end of passage 2, a mean cell harvest of 86.4 ± 38.5 × 10^6^ (mean ± SD) chondrocytes were generated following Quantum^®^ expansion for 8.4 ± 1.5 days, after seeding the bioreactor with 10.2 ± 3.6 × 10^6^ cells (Table 2). Significantly fewer chondrocytes (5.9 ± 4.3 × 10^6^) were expanded from 1.4 ± 0.7 × 10^6^ in 7 ± 2.7 days on TCP with hPL media (*p* = 0.009; paired *t*-test; Fig. 1). In comparison, 1.0 ± 1.1 × 10^6^ chondrocytes seeded on TCP in FBS media yielded 2.3 ± 0.8 × 10^6^ cells after 17.5 ± 21.8 days (Table 2). The number of cells harvested from TCP was not significantly different between chondrocytes cultured in either hPL or FBS at both passage 1 and 2 (Fig. 1A, B).

When considering the differing starting numbers of chondrocytes on TCP (0.4–2.1 × 10^6^) compared with Quantum^®^ (5.0–15.0 × 10^6^), the % cellular increase was not significantly different between the TCP and Quantum hPL expansion (*p* = 0.13; paired *t*-test; Fig. 1).

**FIG. 2.**
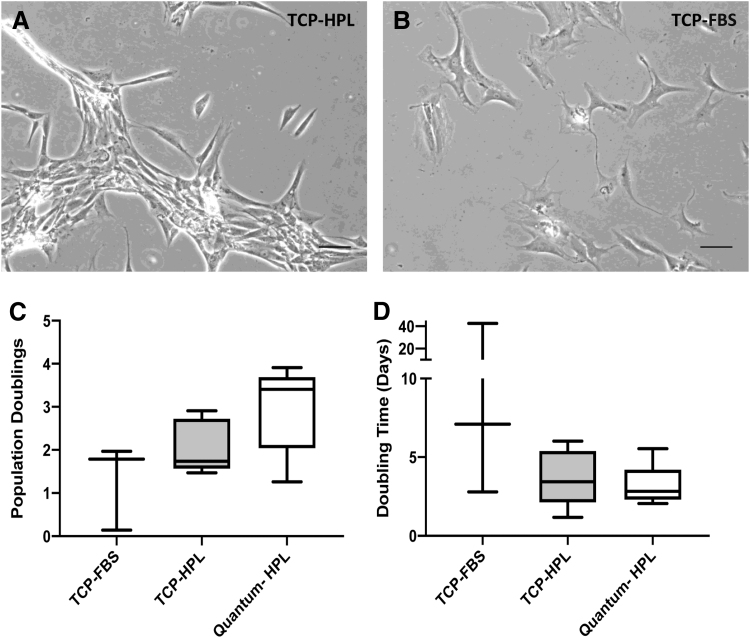
Cell morphologies and growth kinetics of chondrocytes expanded in the Quantum^®^ bioreactor in hPL and on TCP in hPL and FBS. Phase-contrast images of chondrocytes cultured in **(A)** hPL or **(B)** FBS at passage 1. Scale bar represents 100 μm. Neither the **(C)** number of population doublings or **(D)** the population doubling time, at passage 1–2, was different between chondrocytes cultured in the Quantum^®^ bioreactor cf. TCP in hPL or FBS nor between the matched TCP-cultured (hPL cf. FBS) chondrocytes (paired *t*-test; *p* > 0.05). Data are mean ± standard deviation.

Chondrocytes maintained comparative DTs in the Quantum^®^ compared with matched hPL TCP sister populations (Fig. 2D; *p* = 0.30; paired *t*-test). The total population doublings was higher but not significantly increased following Quantum^®^ expansion when compared with TCP (Fig. 2C; *p* = 0.06; paired *t*-test). Moreover, the number of population doublings and the DTs was not significantly higher when comparing matched FBS and hPL cultures on TCP (Fig. 2C, D; population doublings: *p* = 0.26; DT: *p* = 0.35; paired *t*-test). Culture in hPL resulted in a different cell morphology compared with standard culture in FBS, with cells demonstrating more clustered, fibroblast-like growth formations (Fig. 2A, B).

### Immunoprofiling

Chondrocytes cultured in hPL on TCP or in the Quantum^®^ or in FBS adhered to the International Stem Cell Therapy (ISCT) minimal reporting criteria^17^ for being >95% positive for CD105, CD73, and CD90 and negative (<2%) for CD34, CD45, and CD19. However, chondrocytes were immunopositive for CD14 regardless of the expansion method used (TCP hPL: 17.2 ± 14.6; TCP FBS: 25.8 ± 24.4; Quantum^®^ hPL: 25.1 ± 29.3; mean ± SD).

**FIG. 3.**
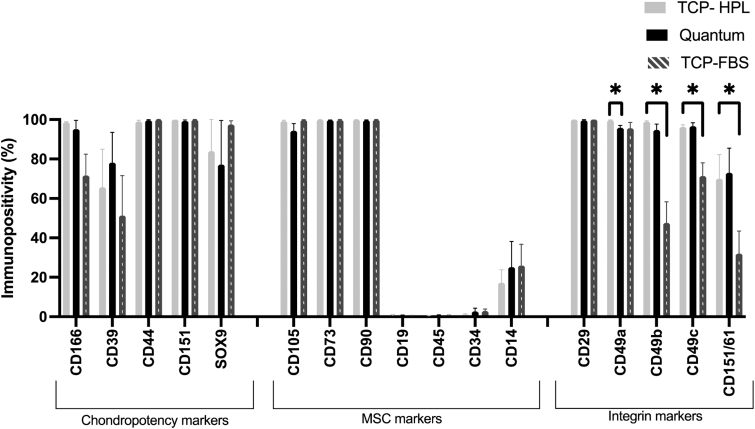
Chondrocyte characterization using flow cytometry following passage 1. Chondrocytes cultured in hPL either in the Quantum bioreactor or on TCP were not differentially immunopositive for chondropotency or MSC markers. CD49a was higher following culture in hPL on TCP cf. in the Quantum^®^ bioreactor (*p* < 0.05; paired *t*-test). Chondrocytes cultured on TCP in either hPL or FBS were not differentially immunopositive for chondropotency or MSC markers. Immunopositivity for CD49b, CD49c, and CD151/61 was higher following culture in hPL cf. FBS on TCP (*p* < 0.05; paired *t*-test). MSC, mesenchymal stromal cell.

Quantum^®^ expansion did not result in altered expression of chondropotency indicators (CD markers 166, 39, 44, 151, SOX9) or MSC profile indicators (CD105, CD73, CD90, CD19, CD45, CD34, CD14) (Fig. 3; *p* > 0.05; paired *t*-test). One of the integrin markers, CD49a (integrin alpha-1), was found to be significantly lower in Quantum^®^-expanded chondrocytes compared with matched cells grown on TCP in hPL (Quantum = 95.8 ± 2.9%, TCP = 99.6 ± 0.4%; mean ± SD; *p* = 0.04; paired *t*-test).

The culture of chondrocytes in hPL demonstrated equivalent immunopositivity for the panel of chondropotency markers and MSC markers tested, compared with matched chondrocytes cultured in FBS (Fig. 3; *p* < 0.05; paired *t*-test). However, the immunopositivity of a number of integrin markers was lower following expansion in FBS compared with hPL (Fig. 3). These include CD49b (integrin alpha 2), which was 99.0 ± 0.8% positive on chondrocytes cultured in hPL and was 47.5 ± 24.1% positive following FBS expansion (*p* = 0.02; paired *t*-test). Furthermore, CD49c demonstrated 96.2 ± 2.6% immunopositivity following hPL expansion cf. 71.2 ± 15.1% FBS expansion (*p* = 0.02; paired *t*-test). CD151/61 positivity was decreased following FBS expansion compared with hPL (FBS = 69.9 ± 27.6%; hPL = 31.9 ± 25.8%; *p* = 0.02; paired *t*-test).

### Chondrogenesis

**FIG. 4.**
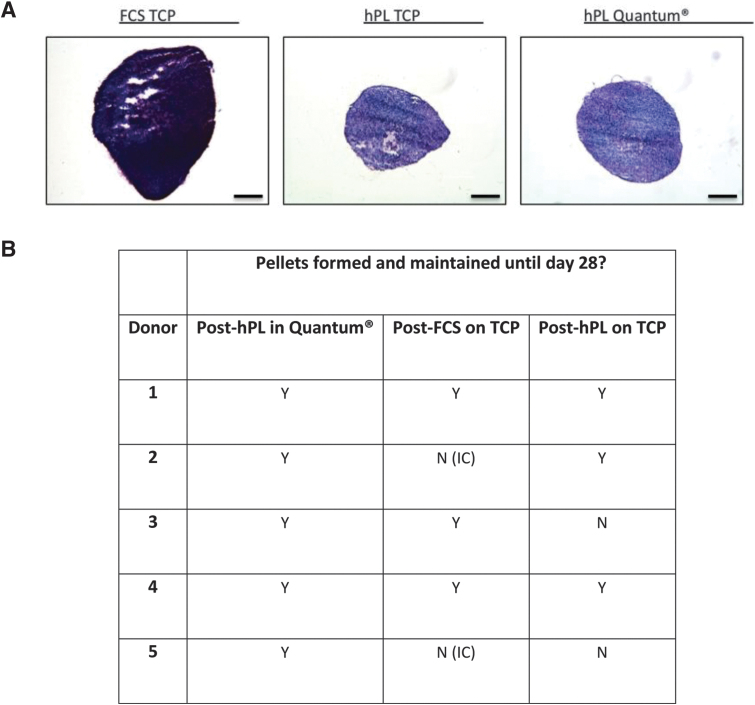
**(A)** Representative images of chondrogenic pellets cultured from chondrocytes following culture in hPL either in the Quantum^®^ bioreactor or on TCP and in FBS on TCP. Chondrogenic pellets were stained using toluidine blue to assess glycosaminoglycans. Scale bars represent 100 μm. **(B)** For two donors, chondrogenic pellets could not be formed or maintained in hPL until 28 days, as demonstrated in the table; Y = yes and N = no. For another two donors, there were IC harvested following FBS expansion to set sufficient numbers of chondrogenic pellets for analysis. IC, insufficient cells.

Following TCP expansion in hPL or FBS and Quantum^®^ bioreactor expansion in hPL, chondrogenic pellets were established and maintained in chondrogenic media for 28 days. There were two donors in which chondrocytes were set for pellet expansion in hPL, but a chondrogenic pellet did not form; rather a cell pellet collected within the Eppendorf tube following centrifugation, but the pellet did not form a sphere over the first few days and once the pellet was dislodged after 3 days, the cells dispersed throughout the media. Furthermore, there were two donors for which there were insufficient cells harvested at the end of passage 1 to set chondrogenic pellets for analysis, once cells had been utilized for DNA and flow cytometry analysis. The conditions for which pellets were formed and maintained to 28 days are demonstrated in Figure 4B.

**FIG. 5.**
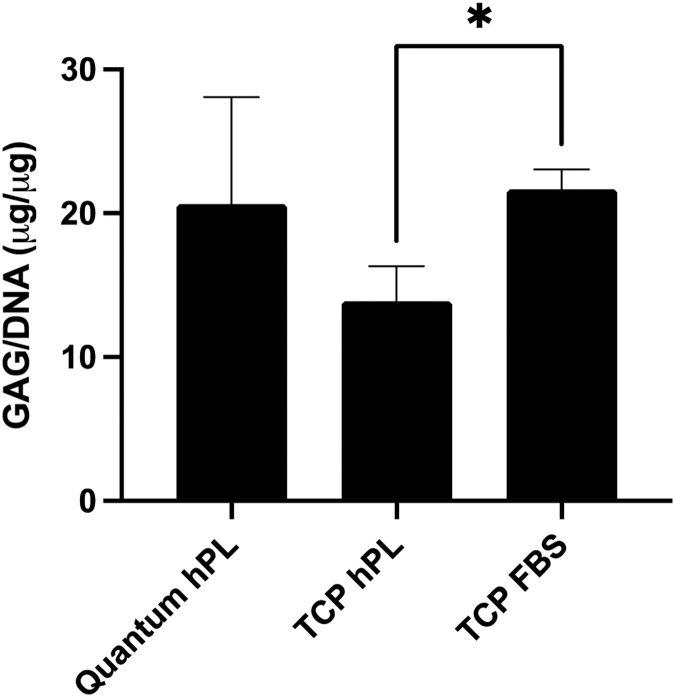
Chondrogenic assessment of pellet cultures following expansion in the Quantum^®^ bioreactor or on TCP in hPL or on TCP in FBS. GAGs were measured after chondrogenic differentiation using the DMMB assay and normalized to the DNA content of pellets. Pellets cultured on TCP demonstrated decreased GAG/DNA content following expansion in hPL media compared with FBS media (Kruskal–Wallis; *p* < 0.05). DMMB, dimethyl blue; GAGs, glycosaminoglycans.

The concentration of chondrogenic pellet GAG content was calculated and normalized to DNA content. Chondrogenic pellets that were formed from Quantum^®^-expanded chondrocytes retained consistent GAG/DNA concentrations when compared with matched sister populations of chondrocytes cultured in hPL on TCP (*p* > 0.05; Kruskal–Wallis; Fig. 5). However, Figure 5 demonstrates that when comparing the matched TCP cultures, expansion in FBS resulted in significantly increased GAG/DNA content in comparison with hPL expansion (*p* > 0.05; Kruskal–Wallis).

### Analysis of telomere length

**FIG. 6.**
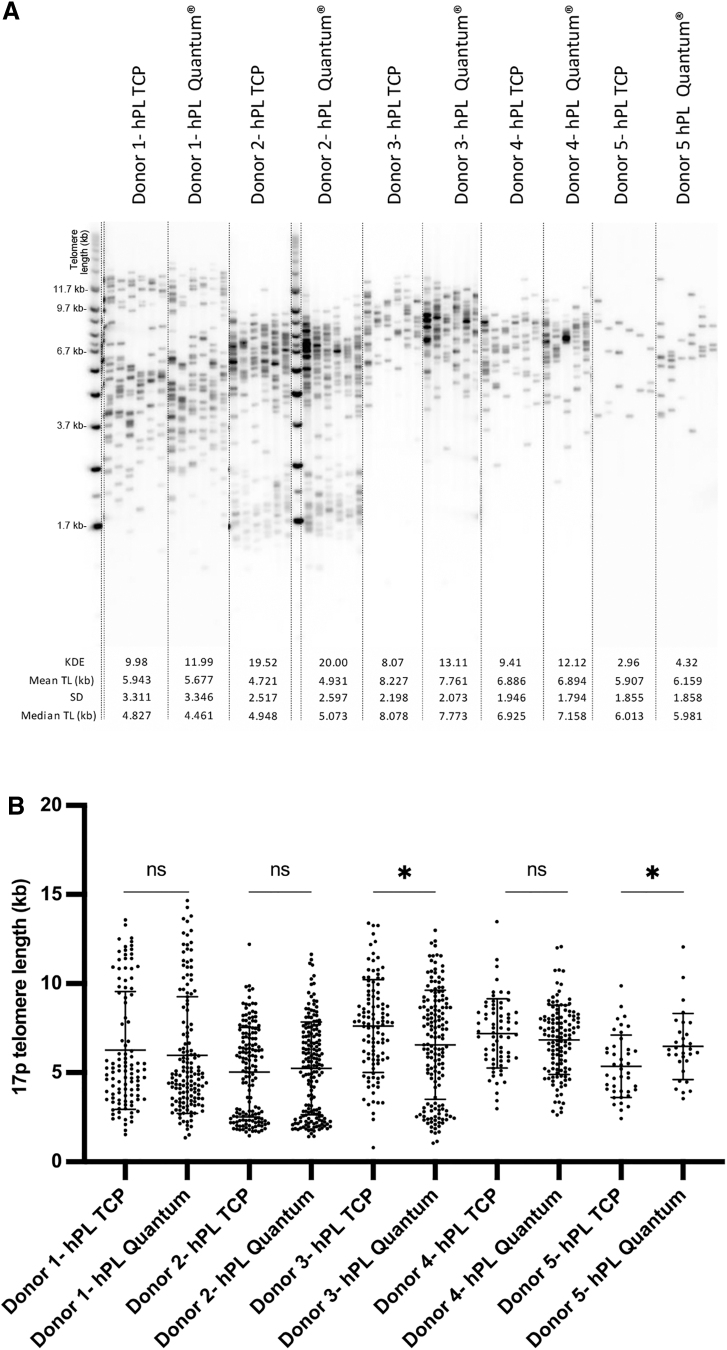
STELA of the 17p telomeres. **(A)** Gels for matched samples cultured on TCP or in the Quantum^®^ bioreactor in hPL. **(B)** Mean telomere length (kb) was not consistently altered between matched donors cultured on TCP cf. Quantum^®^, with only one donor demonstrating decreased telomere length (donor 3) and the other having increased telomere length (donor 5) following Quantum^®^ expansion (*p* < 0.05; Mann–Whitney). STELA, single telomere length analysis.

STELA of 17p telomeres demonstrated that there was a bimodal distribution for many of the chondrocyte samples. In general, this distribution was maintained whether expanded on TCP or in the Quantum^®^ bioreactor. For three of the donors, there was no difference in telomere lengths between upscale and TCP-expanded chondrocytes (*p* > 0.05; Mann–Whitney). However, for two donors, there was a difference in telomere length when expanded in the Quantum^®^ bioreactor in comparison with matched TCP (Fig. 6B). The direction of change, however, was inconsistent for these two donors, with donor 3 demonstrating decreased and donor 5 displaying increased 17p telomere length following Quantum expansion cf. matched hPL expansion on TCP (Fig. 6B) (*p* < 0.05; Mann–Whitney).

## Discussion

The development of allogeneic chondrocyte therapies is a rapidly advancing field.^25,26^ However, it is recognized that the translation of these allogeneic therapies into clinical practice has been limited, likely due to difficulties in sourcing appropriate adult articular cartilage, growing sufficient numbers of cells under GMP conditions, and difficulties with preserving cells appropriately.^27^ As part of developing allogeneic chondrocyte therapies, there is a need to optimize existing autologous chondrocyte manufacturing processes, particularly to grow large numbers of cells from a cartilage harvest biopsy, with standard ACI chondrocyte expansion on TCP for 2–3 weeks^6,28^ unlikely to be capable of producing sufficient cell yields to treat more than a single patient.

In this study, we have presented what we believe to be the first attempt to manufacture chondrocytes in the GMP-compliant Quantum^®^ bioreactor. Although FBS is used in current ATMP manufacture,^6,29^ there is an aim to minimize the use of xeno-products in ATMP manufacture, in accordance with the Note for Guidance on Minimizing the Risk of Transmitting Animal Spongiform Encephalopathy Agents via Human and Veterinary Medicinal Products (EMA/410/01 rev 3).^30^ Therefore, we have aimed to “future-proof” this study by investigating pooled hPL as a GMP-compliant, xeno-free alternative^31^ to FBS and testing its potential for use in upscale bioreactor manufacturing. Currently, autologous serum is used for autologous chondrocyte manufacture; this would not be available from an allogeneic donor, particularly in the volumes required for bioreactor expansion. Furthermore, the Quantum^®^ expansion products and TCP “sister” populations have been extensively characterized.

Our findings demonstrate that chondrocytes can be successfully maintained and expanded using the Quantum^®^ bioreactor system. Manufacture in this advanced culture platform should be easily transferrable to a GMP facility and so significantly reduce the need for “open-handling processes,” in which there is increased potential for culture contamination.^32^ Moreover, we have highlighted that large quantities of chondrocytes (mean = 86 M) can be manufactured in a single passage in this system so as to maximize the cell yields from donor tissues. Currently patients treated with ACI in our center have 1–16 M cells delivered into a defect with a diameter of 20.7 ± 7.5 mm (mean ± SD; *n* = 306; unpublished data from our onsite GMP facility). With this in mind, our data indicate that an average Quantum^®^ yield could potentially produce up to 84 batches of chondrocytes from a single expansion phase.

In developing cell-based therapies, staffing and consumables are the two biggest associated costs.^33^ Quantum^®^ bioreactor expansion of chondrocytes has the potential to significantly reduce costs in-terms of expert staffing, with five staff having been proposed to be required to harvest the number of cells that one user could harvest from the Quantum^®^.^32^ However, the consumables required for Quantum manufacture are expensive and would need to be considered before an accurate cost/benefit could be determined, as has been performed for other cell types.^32,34^

Importantly, chondrocytes manufactured in the Quantum^®^ retained many of the characteristics of matched cultures grown on TCP. This included the immunopositivity of chondropotency and MSC markers postexpansion and capacity for producing chondrogenic pellets. Importantly, chondrocytes retained high levels of CD44 and CD166. Surface expression of CD44 on chondrocytes used for ACI is associated with improved clinical outcomes (International Knee Documentation Committee score^35^ and Lysholm score at 24 months^15^). Higher CD44 chondrocyte expression has also been demonstrated to correlate with chondrocyte capacity to form GAGs.^16^ Furthermore, increased expression of CD166 on chondrocytes has been shown in groups with enhanced clinical success following ACI,^15^ as well as having increased expression during chondrogenic redifferentiation.^36^

Immunoprofiling of chondrocyte products from each culture condition indicates that these cells will have the capacity to repair cartilage, however, future work would further benefit from analysis of additional chondrocyte markers such as collagen type II^35^ and aggrecan, as alternative markers of chondrocyte potency. On the whole the integrin profiles were comparable between Quantum^®^- and hPL TCP-expanded chondrocytes, with the exception of CD49a (integrin α1), which was reduced post-Quantum^®^ expansion. The alpha 1 subunit makes up half of the α1β1 integrin, which is expressed on normal chondrocytes and binds to collagen types VI and II and also to matrilin-1.^37^ Djouad et al. demonstrated that gene expression of integrin α1 increases over time as MSCs undergo chondrogenesis.^38^ This may perhaps indicate that the chondrocytes are starting to dedifferentiate following Quantum^®^ expansion, but this would require confirmation at the gene expression level.

Telomere lengths were not altered following upscale expansion of the chondrocytes, indicating that cellular aging was not induced as a response to rapid expansion.^39^ These findings suggest that Quantum^®^ bioreactor expansion could provide a safe method of producing large numbers of chondrocytes without detrimentally aging the chondrocytes. It will be important, however, to determine whether the capacity of these chondrocytes for repairing cartilage is maintained in *in vivo* models of pre/early-OA, particularly as studies have indicated that the potency of chondrocytes as assessed via immunoprofile and gene expression relationship with chondrogenic pellet assays does not always relate to clinical outcomes.^15^

Interestingly, chondrocytes manufactured in hPL resulted in greater cell yields, as normalized to seeding density, with a much quicker DT than those cultured in FBS. Sykes et al. also demonstrated that chondrocytes manufactured in hPL had an increased rate of proliferation when compared with manufacture in FBS. Akin with our findings, chondrogenic pellets derived from hPL-manufactured chondrocytes demonstrated reduced chondrogenicity (GAG content normalized to cell number) and formed less stable pellets.^40^ Chondroprogenitors, a subpopulation of fibronectin-adherent proliferative chondrocytes, expanded in hPL had decreased gene expression of chondrogenic markers (aggrecan and collagen II).^41^ Conversely, Rikkers et al. demonstrated increased GAG content in 28-day chondrogenic pellets established from chondrocytes expanded in hPL compared with those grown in FBS.^42^ However, when redifferentiated in the presence of hPL in the chondrogenic differentiation media, matrix production and chondrogenic gene expression were negatively influenced.^42^ When seeded in fibrin scaffolds, however, chondrocytes differentiated using media supplemented with hPL produced higher GAG content compared with standard chondrogenic differentiation media and demonstrated a trend toward increased gene expression of chondrogenic markers (aggrecan, collagen type II alpha 1 chain, and cartilage oligomeric protein).^43^

All these data suggest that hPL results in greater numbers of cells in a given time period. How hPL influences their capacity to form cartilage extracellular matrix, however, remains less conclusive with inconsistent findings across studies. Future studies are required to assess how different hPL concentrations and from different suppliers influence chondrocyte potency to determine if suboptimal chondrogenic differentiation is a direct influence of the quality of the hPL being used for their expansion.

It is important to note that for many current clinical manufacturing processes, human autologous serum is used for chondrocyte expansion.^2,6,28^ A recent study has compared chondrocyte expansion in autologous serum and hPL.^44^ Philippe et al. demonstrated that human articular chondrocytes maintained similar morphology and growth kinetics when cultured in hPL compared with autologous serum and indicated that when these hPL-grown chondrocytes were cultured to form chondrogenic pellets, they accumulated GAG and demonstrated increased chondrogenic gene expression increasingly with time throughout their culture. A limitation of this study was that we unfortunately did not have sufficient cells to derive enough chondrogenic pellets such as to harvest them for analysis at time points throughout the 28-day culture period, although it would certainly have been interesting to determine how the chondrogenic potential compared longitudinally between chondrocytes manufactured in hPL and FBS.

Furthermore, the variability between batches of hPL, even from commercial, GMP-compliant sources, is more recently becoming acknowledged.^45^ In an attempt to limit this effect across the different donor sources, the same batch of hPL was used in all experiments. In future work, however, it would be important to ensure that the hPL source adheres with the recommendations of the Working Party for Cellular Therapies of the International Society of Blood Transfusion.^45^

To date, several studies have manufactured a plethora of cell types in the Quantum^®^, using hPL (5–10%) as a growth supplement. This has included numerous studies that have upscaled MSCs from various sources, including adipose tissue^46–48^ and bone marrow.^5,32,34,49–51^ However, only Haack-Sørensen et al. have directly compared Quantum^®^ manufacture in 5% hPL or 10% FBS. This study highlighted that hPL manufacture resulted in an average of 546 million adipose-derived-MSCs in 9 days compared with 111 million cells in 17 days following FBS manufacture.^46^ Moreover, these cells maintained equivalent quality in terms of adherence with the ISCT criteria and genomic stability.^46^ As our study results also indicate slower chondrocyte growth in FBS cf. hPL on TCP, a study of Quantum^®^ chondrocyte expansion in FBS to assess yields, DTs, and chondrogenic potential is warranted.

Although our data indicate that hPL may be suboptimal for chondrocyte manufacture in terms of chondrogenic function, there is a GMP requirement to move away from the use of xeno-sera. Therefore, further investigation of the potential of GMP-compliant defined serum-free media for manufacture of chondrocytes is required. There are several such products commercially available, but the majority have been manufactured for the growth of MSCs and their utility for chondrocyte expansion without encouraging dedifferentiation needs to be comprehensively evaluated. Moreover, the paracrine and immunomodulatory function of these Quantum^®^-expanded chondrocytes has not been assessed. Perhaps this activity could prove to influence the repair or regenerative properties of chondrocytes in much the same way as appears to be the case for MSCs.^52,53^

Alongside optimization of manufacturing procedures for allogeneic chondrocyte therapies, there is a need to identify optimal donor sources of cartilage. This study highlights that chondrocytes can be upscale expanded within the Quantum^®^ bioreactor system. However, the use of chondrocytes, which have been derived from joint arthroplasty tissue, is unlikely to be the best source of cartilage for clinical use, as the extracted chondrocytes may have been detrimentally preconditioned within the osteoarthritic joint. Therefore, identification of optimal “healthy” donors is required, perhaps from cadaveric adult cartilage donors, akin to clinical allograft sources. Alternatively, more proliferative chondrocyte populations, such as those of chondroprogenitors, which can be isolated via selective adhesion to fibronectin^54,55^ or from juvenile tissue sources, such as excised polydactyl digits,^56,57^ may be superior.

Moreover, before chondrocytes from any of these donors could be used in clinical practice, a concerted effort is required to ensure that all the manufacturing steps comply with GMP regulations and that rigorous safety testing of the cell products, for example, donor serological testing and product microbiology, endotoxin and mycoplasma tests, is performed. Furthermore, to confirm that minimum batch-to-batch functionality is achieved, a potency release test would need to be established. Regardless, a method of assessing and scoring the quality of the donor cartilage tissue and extracted chondrocytes will be a requisite before costly upscale bioreactor expansion is performed.

Cartilage repair therapy has been at the forefront in introducing advanced therapies into the clinic, with ACI having been applied for more than 30 years.^1,58^ Furthermore, ACI was one of the pioneering cell therapy techniques to be recommended by the UK National Institute of Clinical Excellence.^59^ As cell therapy for cartilage repair continues to evolve, there is a need to manufacture large numbers of chondrocytes for “off-the-shelf” delivery. The findings of this study indicate that there is potential to upscale expand chondrocytes using the Quantum^®^ bioreactor. With refinement, bioreactor expansion of allogeneic chondrocytes could result in a relatively cheap and consistent ATMP with the potential to repair cartilage and prevent OA in many patients.
